# Green dialysate and gallbladder perforation in a peritoneal dialysis patients: a case report and literature review

**DOI:** 10.1186/s12882-018-0974-6

**Published:** 2018-07-04

**Authors:** Yueh-Lin Wu, Yi-Sheng Lin, Thomas Yu-Ren Hsueh, Wen-Ching Lo, Kuo-Chou Peng, Mu-Jung Kao

**Affiliations:** 1 Department of Nephrology, Taipei City Hospital, Zhongxiao Branch, No. 87, Tongde Road, Nangang District, Taipei, 115 Taiwan; 2 Division of Urology, Taipei City Hospital, Ren-Ai Branch, Taipei, Taiwan; 3 Division of Gastroenterology, Taipei City Hospital, Zhongxiao Branch, Taipei, Taiwan; 4 Division of Medical Imaging, Taipei City Hospital, Zhongxiao Branch, Taipei, Taiwan; 5 Division of Rehabilitation, Taipei City Hospital, Zhongxiao Branch, Taipei, Taiwan

**Keywords:** Gallbladder perforation, Peritoneal dialysis, Green dialysate, Cholecystectomy, Peritonitis

## Abstract

**Background:**

Gallbladder perforation is a rare but lethal condition and its diagnosis is usually difficult and delayed. Frequently, gallbladder rupture is associated with cholecystitis, but spontaneous perforation was ever described. However, spontaneous rupture of gallbladder has never been reported in patients underwent peritoneal dialysis.

**Case presentation:**

We report a 62-year-old man who presented with abdominal pain for 2 days to clinic. Peritoneal dialysis-related peritonitis was diagnosed initially. It was followed by spontaneous gallbladder perforation with greenish dialysate. The patient was managed successfully by antibiotic treatment and primary closure of gallbladder perforation with external drainage. He recovered from this critical condition and stayed on dialysis.

**Conclusions:**

Early diagnosis and timely surgical intervention yields a good prognosis in PD patients with gallbladder perforation. Surgical intervention and antibiotic treatment are the mainstay of treatment. Both of them should take place promptly.

## Background

Gallbladder perforation is an uncommon but lethal condition of gallbladder disorder which its diagnosis is often delayed [1]. Gallbladder perforation is frequently associated with acute cholecystitis and gallbladder stones [2, 3] but can also occur spontaneously in rare conditions [4]. There is a high prevalence of gallstone disease in dialysis patients [5]. These patients also have a higher risk for acute cholecystitis, compared with patients not on dialysis [6]. Among dialysis patients with acute cholecystitis, patients on peritoneal dialysis (PD) have higher mortality rates [6]. The reports on gallbladder perforation among PD patients, however, are limited. To our knowledge, no spontaneous gallbladder perforation in a patient underwent PD has never been reported. Here, we report the first case of spontaneous gallbladder perforation in a PD patient who was managed successfully without cholecystectomy. In addition, we performed a literature review to further discuss the important aspects of presentation, diagnosis and management.

## Case presentation

A 52-year-old man with a history of hypertension, coronary artery disease and end-stage renal disease under continuous ambulatory PD treatment for 3 years presented to the PD clinic with cloudy dialysate effluent and diffuse abdominal pain lasting 2 h. Two weeks before this presentation, he was diagnosed with community-acquired pneumonia, which was treated with 400-mg oral moxifloxacin daily for 10 days. However, persistent intractable cough did not seem to improve. Two days before this presentation, he visited the emergent department due to epigastric pain and an intractable cough. He had no nausea, vomiting or fever. Physical examination showed epigastric tenderness without guarding and rebound pain. Laboratory studies indicated a white blood cell (WBC) count 12,000 cells/mm^3^ with 87% neutrophils and 8% lymphocytes, hemoglobin 8.0 g/dL, alanine transaminase 35 U/L, amylase 19 U/L, total bilirubin 0.56 mg/dL, creatinine 16.7 mg/dL, sodium concentration 133 mEq/L and potassium concentration 4.2 mEq/L. Chest radiography indicated no signs of pneumonia. PD dialysate effluent analysis revealed WBC 2/μL without polymorphonuclear leukocyte (PMN). Abdominal ultrasonography only showed a distended gallbladder with sludge. Severe strains of abdominal muscles from persistent and intense cough were impressed. He was discharged with antitussives and analgesics.

**Fig. 1:**
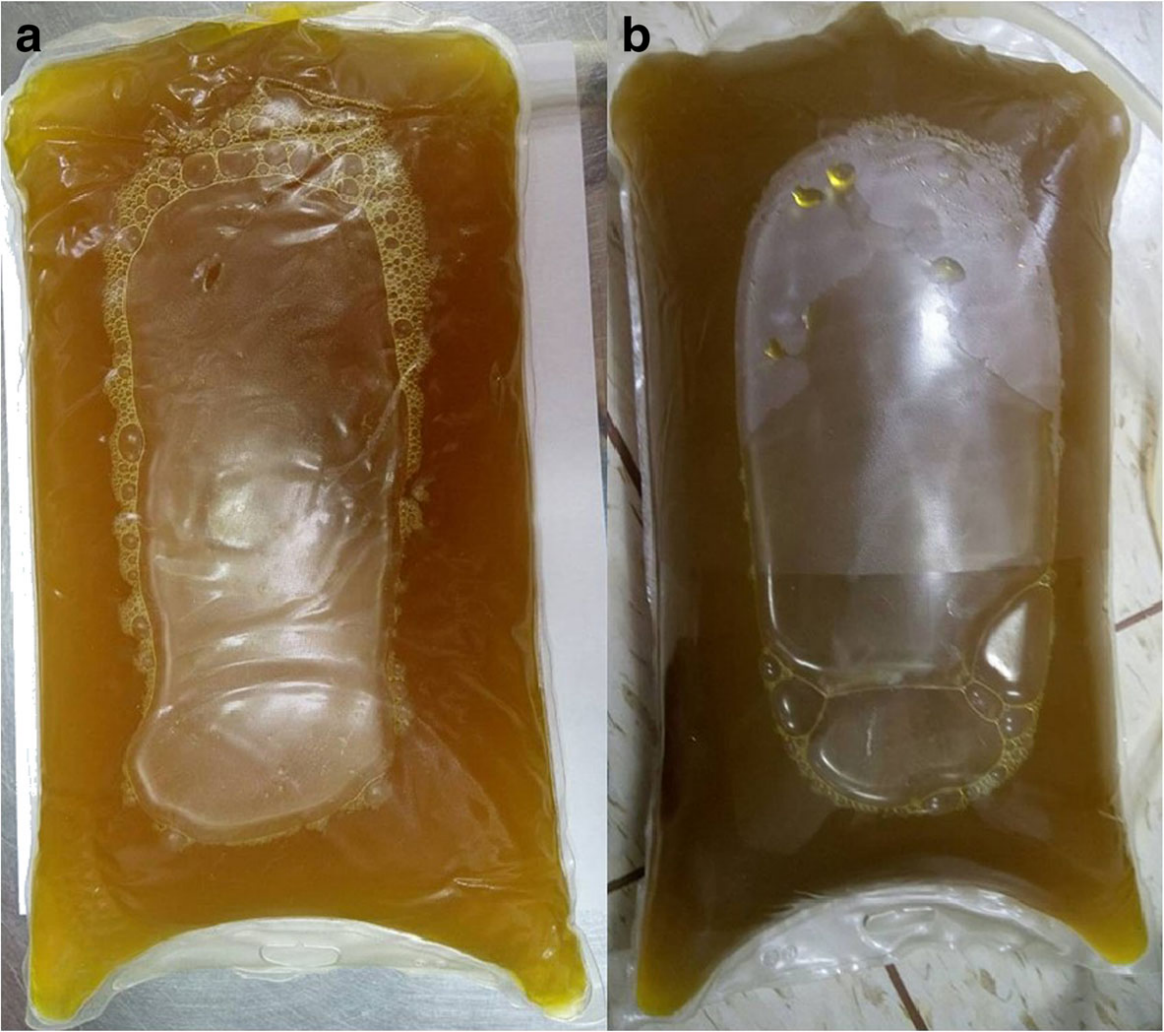
Peritoneal dialysis fluid. The color of peritoneal dialysis fluid was from yellow at initial presentation (**a**) and became green after fasting for 24 h (**b**)

**Fig. 2:**
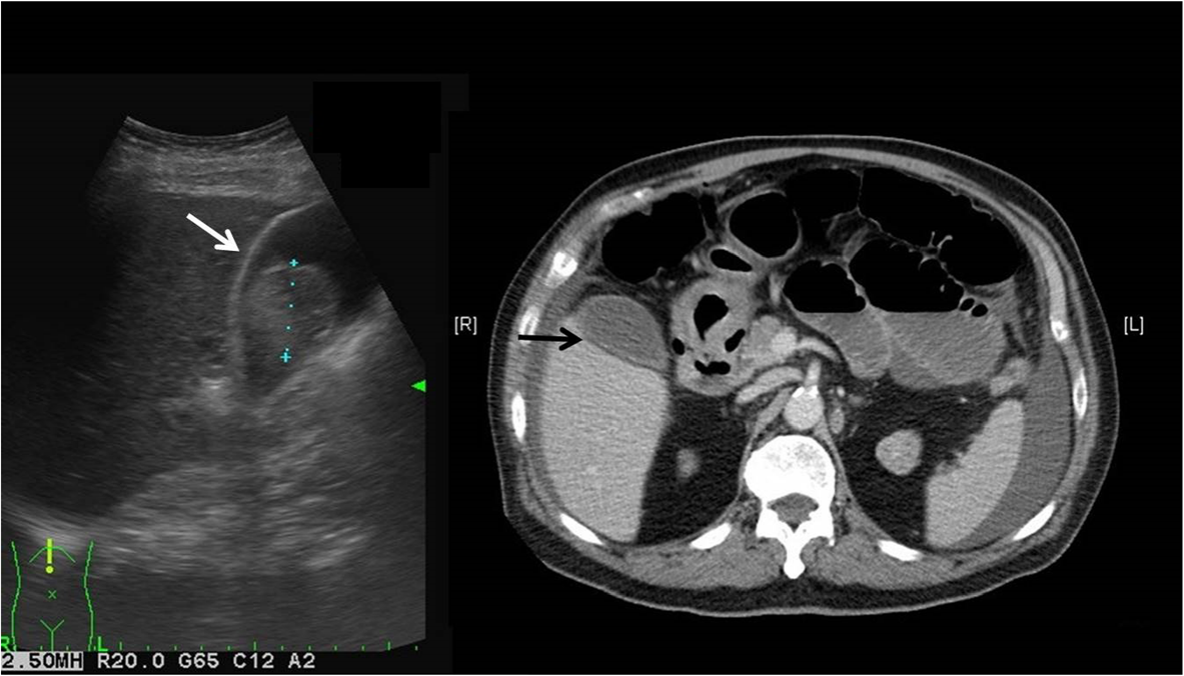
Abdominal ultrasonography revealed distended gall bladder with sludge within it without wall thickening (white arrow); Abdominal computed tomography scan revealed dilated bowel with air-fluid level, much fluid around spleen and a distended gallbladder with modest thickening of the wall (black arrow)

At the PD clinic, his vital signs were body temperature 36 °C, heart rate 104 per minute, respiratory rate 19 per minute and blood pressure 121/91 mmHg. Physical examination confirmed diffuse abdominal tenderness with peritoneal irritation and clean exit site of PD catheter. PD dialysate effluent analysis revealed WBC 1783/μL including 50% PMN. He was diagnosed with PD peritonitis and admitted to ward. Cefuroxime and amikacin were administered intraperitoneally empirically. He had diffuse abdominal pain, nausea and vomiting. Daily analysis of PD dialysate effluent showed WBC counts of 1565/μL(PMN 71%) on day 2 and 3755/μL (PMN: 66%) on day 3 and 4805/μL (PMN:86%) on day 4. PD effluent culture was positive for *Klebsiella pneumoniae* on day 3. Cefuroxime and amikacin were replaced by Cefepime intraperitoneally. Owing to persistent symptoms, we repeated dialysate culture on Day 3 and it grew yeast-like organism on day 6. Amphotericin was administered immediately and surgery to remove the PD catheter was scheduled. On the same day, dark yellow dialysate was obtained (Fig. 1a). The color became green after a 24-h fast (Fig. 1b). An enhanced computed tomography (CT) scan of abdomen only revealed a distended gallbladder with modest wall thickening and distended bowel (Fig. 2).

Exploratory laparotomy was performed on day 8. Despite a thorough examination of the intestine and stomach, no perforation was identified. The gallbladder was neither reddish nor edematous. A 0.3-cm rupture was found on the fundus of gallbladder. Primary closure of gallbladder was done with external drainage. The PD catheter was removed uneventfully. After antibiotic treatment and intensive care, he recovered without sequelae. He was discharged after 37 days hospitalization and received maintenance hemodialysis three times a week.

## Discussion

Gallbladder perforation is a rare but potentially fatal condition [1]. Gallbladder perforation is most often associated with acute cholecystitis and gallbladder stones [2, 3]. It may also occur spontaneously as well as a complication of malignancy, medical instrumental examinations or abdominal trauma [4, 7, 8]. Gallbladder stone disease is a frequent condition which affects up to 28% of the dialysis patients [5]. The incidence rate of acute cholecystitis in the dialysis patients (5.8 per 1000 patient-years) is substantially higher than it in general population (0.92 per 1000 patient-years) [6]. Accordingly, dialysis patients might have a higher risk for gallbladder perforation. Due to inadequate diagnosis and delayed surgery causing high mortality of gallbladder perforation, the presentation, diagnosis and management of gallbladder perforation in PD patients are worthy of discussion. Herein, we presented a case of spontaneous gallbladder perforation in a patient on chronic PD and analyzed retrospectively the other six PD patients with gallbladder perforation in the past literature (Table 1) [9–14].

**Table 1:** Characteristics of peritoneal dialysis patients with gallbladder perforation

Reference	Geddes et al., 1996 [9]	Babin et al., 2006 [10]	Chen et al., 2010 [11]	Gobel et al., 2011 [12]	Chao et al., 2012 [13]	Silda et al., 2016 [14]	Present report
Age	51	53	80	30	81	81	52
Gender Male	M	M	M	F	F	M	M
Dialysis Vintage	7 m	8 y	2 m	7 y	2 y	3 y	3 y
Renal disease	ARVD	T1DM	HT	HUS, RCC s/p Nx	CHF	CIN	T2DM, CAD
Symptoms^a^	**+**	**–**	**+**	**+**	**–**	**–**	**+**
GB stones	**–**	**–**	**+**	**+**	**–**	**+**	**–**
Culture of dialysate	Enterococcus *dirans*	Negative × 2	NA	*Escherichia coli*, Candida *glabrata*	NA	Escherichia coli	Enterococcus *faecium*, *Candidatropicalis*,
Presence of green dialysate	on day 14	on admission	on admission	No green dialysate	on admission	on day 10	on day 6
Green dialysate to surgery (days)	2	2	urgent	–	1	2	2
Intervention	Laparotomy + OC	Laparoscopy + OC	OC	OC + debridement	OC	OC	Closure of perforation
Diagnosis	Acute cholecystitis with GB perforation	Acute necrotizing cholecystitis with GB perforation	Transmural necrotic gallbladder	Chronic cholecystitis with focal perforation	Gangrenous cholecystitis with wall leakage	Phlegmonous cholecystitis with micro-perforations	Spontaneous GB perforation
Perforation type	I	I	I	II	I	I	I
Outcomes	Recovery	Recovery	Recovery	Recovery	Respiratory care ward	Recovery	Recovery
Dialysis outcome	Shift to long-term HD	Temporary HD for 28 days, Keep in PD	Shift to long-term HD	Shift to long-term HD	NA	Temporary HD for 14 days, Keep in PD	Shift to long-term HD

*ARVD* atherosclerotic renovascular disease, *T1DM* type 1 diabetes mellitus, *HT* Hypertension, *HUS* hemolytic-uremic syndrome, *RCC* Renal cell carcinoma, *Nx* Nephrectomy, *CHF* congestive heart failure, *CIN* chronic interstitial nephritis, *T2DM* type 2 diabetes mellitus, *NA* not available, *OC* Open cholecystectomy, *GB* gallbladder, *HD* hemodialysis, *PD* peritoneal dialysis

^a^symptoms: fever or abdominal pain

Gallbladder perforation was further classified into three types by Niemeier in 1934 [15]. Type I is an acute free perforation into the peritoneal cavity, type II a subacute perforation with pericholecystic abscess formation, and type III a chronic perforation with cholecystoenteric fistula [15]. In two previously published large retrospective reviews, the most common gallbladder perforation was type II, ranging from 46.2–52.6% [1, 3] with the mean age of the patients in these studies was approximately 62.1–76.1 years; and the portion of men was 55.4–64.2%; the association between gallbladder perforation and gallbladder stones was about 86.6% [1, 3]. In our current review, however, revealed the gallbladder perforation in PD patients were characterized by two distinct distributions of age in fifties and eighties, more type I perforation (85.7%), significant male predominance (71.4%), and a weaker association between gallbladder perforation and gallbladder stones (42.9%) (Table 1).

More than 70% of patients with gallbladder perforation presented with typical symptom including high fever, nausea, vomiting, and abdominal pain, especially localized to the right upper quadrant area [16–18]. In our literature review, however, we found that more than 40% of PD patients with gallbladder perforation were free from abdominal pain and fever (Table 1). Furthermore, the clinical features suggestive of gallbladder perforation are similar to those of uncomplicated cholecystitis or peritonitis, which are nonspecific [2, 19]. Therefore, gallbladder perforation in PD patients might masquerade as acute cholecystitis, acute pancreatitis, or PD-related peritonitis and be managed conservatively with detrimental consequences [20].

Analysis of PD fluid is a unique and valuable tool for the differential diagnosis in PD patients with acute abdomen. Before the era when sonography and CT scan were widely use, diagnostic abdominal lavage is a sensitive tool for diagnosing acute abdomen and for helping to identify those patients who need further surgical exploration [21]. The process of PD in nature provides a continuous abdominal lavage. The peritonitis can be confirmed by obtaining effluent cell counts, portion of PMNs, and culture reports [22]. Previous literatures also suggest that dialysate cultures of enteric, polymicrobial or fungal organisms are highly suggestive of visceral perforation compared with other types of peritonitis [23–26]. However, culture results can take days, which might not be available or informative during the early period of differential diagnosis. Among the five available culture reports of PD fluid in our review, one had negative a result, two showed polymicrobial organisms, and the others showed enteric bacteria and fungus (Table 1). Elevated amylase or bilirubin levels in PD fluid would also raise the concern for abdominal catastrophe [26]. The level of amylase in PD fluid has been used to distinguish conventional PD-related peritonitis (mean, 11 IU/L; range, 0–90 IU/L) from peritonitis secondary to intra-abdominal pathology (mean, 816; range, 142–1746) [27]. In addition, the elevation of ascetic fluid bilirubin levels in general population indicated ruptured gallbladder or perforated duodenal ulcer [28]. Among these patients, ascitic fluid bilirubin levels greater than 6 mg/dl and an ascitic fluid to serum bilirubin concentration ratio greater than 1.0 were suggestive of gallbladder perforation [28]. Measurement of bilirubin levels in PD fluid has also been used as a bedside tool to help diagnose gallbladder perforation among the PD patients in our current review [9, 10].

One interesting finding from our review is that green dialysate was noted in most patients with gallbladder perforation except for one with type II subacute gallbladder perforation [12]. Moreover, green dialysate can occur in an asymptomatic PD patient of gallbladder perforation without peritonitis [10]. The causes of green or dark yellow dialysate include bile leakage into peritoneum, hyperbilirubinemia, and hemolysis after peritoneal hemorrhage [28, 29]. Bile stained PD fluid can result from ruptured gall bladder, acute pancreatitis or intestinal perforation [28, 30, 31]. Early diagnosis of gallbladder perforation is usually considered difficult. However, the green dialysate effluent provided a very important clue for clinicians. Under the circumstances, it is an important red flag that may quickly help distinguish secondary peritonitis by gallbladder perforation from conventional PD-related peritonitis in PD patients.

A number of publications have addressed the role of imaging studies in diagnosing gallbladder perforation. The direct signs of gallbladder perforation are the visualization of wall defects or demonstration of stones outside the gallbladder [32, 33]. Sonography is the initial mode of investigation on gallbladder pathology. It often fails to demonstrate gallbladder perforation because the examination is always highly operator-dependent and interrupted by increased intestinal gas and pain. Recent studies have demonstrated that the presence of the only reliable sonographic sign, the “hole sign”, for gallbladder perforation is very low, ranging from 0 to 27% [18, 33, 34]. Abdominal CT scan is an invaluable tool in the evaluation of patients with acute abdomen. Not only CT scans can show more indirect indicators of gallbladder perforation including pericholecystic abscess, fluid accumulation and thickening of the wall, but also demonstrate the wall defect directly [32]. Even though CT scans appeared to improve the diagnostic accuracy of gallbladder perforation, the diagnostic rate was still 14.3–50.0% [18, 32, 34]. In the case of spontaneous gallbladder perforation with a mild inflammation status, the preoperative diagnosis is almost impossible because of the absence of typical radiological findings [35, 36]. In this literature review, we found the most common finding in a CT scan was thickening of the gallbladder wall. Except the one patient of type II perforation confirmed by the CT scan with the evidence of gallstone spillage outside the gallbladder, neither sonography nor CT scan showed direct signs of gallbladder perforation in any other patients.

The diagnosis of gallbladder perforation may be difficult based on physical examination, laboratory tests, and imaging studies and may be only established by seeing the perforation intraoperatively or by pathology reports postoperatively [18, 34]. High index of clinical suspicion and early exploratory laparotomy are important factors to improve the outcome of surgical peritonitis in PD patients [26, 37]. Therefore, it is imperative to perform timely exploratory laparotomy and administered broad-spectrum antibiotics in the PD patients with suspected of gallbladder perforation. The initial empiric antibiotics recommended by latest guidelines of PD-related infections from International Society for Peritoneal Dialysis ensure major coverage of enteric bacteria [22]. If visceral perforation is suspected, the choice of metronidazole in combination with ampicillin and ceftazidime or an aminoglycoside is further recommended [22]. Five of the seven patients were initially diagnosed with PD-related peritonitis and administered with intraperitoneal antibiotics. One of the remaining two was diagnosed with type II gallbladder perforation and the other one presented no evidence of peritonitis. Of the five patients who were initially diagnosed with peritonitis, two received urgent open cholecystectomy under the suspicion of gallbladder perforation. The other three with poor response to initial treatment and presence of green dialysate received exploratory laparotomy. It is also possible that perforation had occurred after admission, as an extension of the disease process.

Cholecystectomy, peritoneal lavage, and abscess drainage are preferred treatments for gallbladder perforation [1, 3, 18, 34]. Open cholecystectomy was widely used, whereas laparoscopic cholecystectomy was also advocated for acute, gangrenous and perforated cholecystitis [18, 34]. Laparoscopic approach might not be the suitable alternative in PD patients because the need of highly-experienced surgeons, the difficulty in identification of occult intraabdominal pathology and the high conversion rate to open cholecystectomy (33.3–61.5%) [3, 34]. In the present review, 85.7% of patients received open cholecystectomy while only a patient with spontaneous perforation was successfully managed by primary closure of gallbladder perforation with external drainage. PD catheters were removed from all patients except one was kept intact after an open cholecystectomy [14]. After operation, 2 patients remained on PD after temporary hemodialysis, and the others shifted to long-term hemodialysis.

The mortality of peritonitis due to visceral injury in PD patients could be up to 46.3% [25], and the mortality of gallbladder perforation in general population was approximately 9.5–15.2% [1, 3, 17, 18]. To our surprise, there was no death from gallbladder perforation in the PD patients, who had been considered at higher risk for mortality because of older age and more comorbid conditions. This excellent outcome could be explained that the green dialysate may rise more suspicion and make early diagnosis and timely surgical intervention more possible. The interval between the onset of symptoms and surgery in non-dialysis patients with type I or II gallbladder perforation was around 6.8–7.4 days [18, 34]. In contrast, the duration between the appearance of green or dark yellow dialysate and surgery was usually less than 48 h in PD patients. Furthermore, the PD patients with gallbladder perforation could also benefit from frequent peritoneal lavage by PD fluid exchanges and early administration of empiric antibiotics under the suspicion of PD-related peritonitis at admission [22]. Nephrologists and surgeons are always warned to carefully weigh between the decision to proceed to laparotomy and the complication of surgery. In our review and experience from the current case, when green dialysate is noted and gallbladder perforation is suspected, exploratory laparotomy should be strongly taken into consideration.

## Conclusion

In summary, gallbladder perforation is uncommon in PD patients. Typical symptoms and signs are even less common. The importance of early diagnosis of gallbladder perforation and prompt surgical intervention cannot be overemphasized. Gallbladder perforation diagnosis is always made after exploratory laparotomy. Abdominal CT scans, biochemical tests of PD fluid, and cultures of PD fluid can aid in the diagnosis. We recommend that the presence of green dialysate with or without peritonitis strongly indicates gallbladder perforation. Because of its high mortality and morbidity rate, it is better to perform exploratory laparotomy than to practice watchful waiting when gallbladder perforation is suspected. Open cholecystectomy with PD catheter removal and administration of aggressive antibiotics are preferred treatment. The prognosis of gallbladder perforation in PD patients is good after timely surgical intervention.

## Abbreviations

ARVD: Atherosclerotic renovascular disease
CHF: Congestive heart failure
CIN: Chronic interstitial nephritis
CT: Computed tomography
GB: Gallbladder
HD: Hemodialysis
HT: Hypertension
HUS: Hemolytic-uremic syndrome
NA: Not available
Nx: Nephrectomy
OC: Open cholecystectomy
PD: Peritoneal dialysis
PMN: Polymorphonuclear leukocyte
RCC: Renal cell carcinoma
T1DM: Type 1 diabetes mellitus
T2DM: Type 2 diabetes mellitus
WBC: White blood cell
